# P03-01 The effect of device-free recess on schoolchildren's physical activity and social interaction

**DOI:** 10.1093/eurpub/ckac095.037

**Published:** 2022-08-29

**Authors:** Charlotte Skau Pawlowski, Louise Stjerne Knudsen, Jonas Vesterggard Nielsen, Tanja Schmidt

**Affiliations:** Sports Science and Clinical Biomechanics, University of Southern Denmark, Odense M, Denmark

**Keywords:** schoolchildren, physical activity, social interaction, screen use, device-free recess

## Abstract

**Background:**

In Denmark, 74% of the 11-15-years-old children do not reach national recommendations on physical activity and 6% (∼20.000 children), experience unwanted loneliness. School recess provides a unique opportunity for children to be active with others and form good relations. However, studies have found that many children perceive electronic devices such as mobile phones, tablets and computers as a key barrier for engaging in active play with other children during recess. These findings are based on perceptions from qualitative data. To our knowledge, no studies have investigated the effect of device-free recess on children's recess behaviour. This knowledge is asked for by school boards, health professionals and politicians to inform future policies and actions. Therefore, the aim of this intervention study is to investigate the effect of device-free recess on schoolchildren's physical activity and social interaction.

**Methods:**

During April-June 2020 device-free recess environments will be implemented at seven Danish schools. Children will be asked to place their mobile phones, tablets and computers in locked? device-hotels? during recess for a four-weeks intervention period. Among a cohort of 900 children from Grades 4-6 (10-13 yrs.) physical activity and social interaction during recess will be measured before and during the last intervention week using questionnaire and systematic observation (SOPLAY). All data will be analysedusing multilevel modelling for repeated measures to see pre- versus post intervention changes.

**Results:**

Since the data collection will be carried out in spring 2020, results cannot be presented yet. However, the results will be ready for the HEPA conference in September 2020. Our hypothesis is that the children will increase their physical activity level and social interaction. However, we will also look into recess behaviourpre- and post-intervention across different subgroups of children e.g., gender, age, screen and physical activity habits prior intervention.

**Conclusions:**

The study will be a crucial contribution to the limited knowledge in this field, and in line with the strategy of HEPA, the ambition is to provide evidence-based knowledge on which to base future decisions to improve the everyday conditions for schoolchildren's physical, social and mental development and health.

